# Case report: Safety and efficacy of voxelotor in a patient with sickle cell disease and stage IV chronic kidney disease

**DOI:** 10.3389/fmed.2022.931924

**Published:** 2022-09-15

**Authors:** Awni Alshurafa, Mohamed A. Yassin

**Affiliations:** Hamad Medical Corporation, Doha, Qatar

**Keywords:** sickle cell disease, voxelotor, hemolysis, chronic kidney disease, vaso-occlusive crisis (VOC)

## Abstract

Sickle cell disease (SCD) is a heterogeneous group of inherited disorders characterized by the production of sickle hemoglobin which is less soluble than an adult or fetal hemoglobin. Voxelotor is a hemoglobin S polymerization inhibitor that has been approved for sickle cell disease treatment in the adult and adolescent populations. It acts as a hemoglobin modulator by increasing its affinity to oxygen which prevents red blood cells from sickling. Chronic kidney disease is a common but under-reported complication of SCD and it is a leading cause of morbidity and mortality. The data about the safety and efficacy of voxelotor use in chronic kidney disease is limited. Herein we report a 49-year-old man, with sickle cell disease and stage IV chronic kidney disease, who was managed successfully with voxelotor and resulted in decreasing transfusion requirement and vaso-occlusive painful crisis without affecting kidney function.

## Introduction

Sickle cell disease (SCD) is a heterogeneous group of inherited disorders characterized by the production of sickle hemoglobin, which polymerizes when deoxygenated resulting in rigid and very fragile red blood cells. Hemoglobin SS is the most common genotype, however, other genotypes like C, D, and E present with different phenotypes of chronic hemolysis and recurrent vaso-occlusive crisis (1, 2). SCD affects millions of people worldwide. It is estimated that the number of patients with SCD in the United States may approach 100,000 (3, 4). SCD is a multi-organ disorder that is associated with high morbidity and mortality. The major clinical features are chronic hemolysis, infection, vaso-occlusive events that cause pain, tissue ischemia, and sometimes infarction (5, 6).

Voxelotor is a hemoglobin S polymerization inhibitor that has been approved for sickle cell disease treatment by the United States Food and Drug Administration (FDA) November 2019 for the adult and adolescent population (≥12 years) (7). It reversibly binds to hemoglobin and increases its affinity to oxygen, stabilizing sickle hemoglobin and preventing polymerization. It can be considered for patients who did not tolerate hydroxyurea or can be prescribed as an additional treatment for patients who are refractory on maximum doses of hydroxyurea. The most common adverse effects are headache, abdominal pain, nausea, skin rash, and fever (8).

Chronic kidney disease is a common but under-reported complication of SCD and is a leading cause of morbidity and mortality. The annual incidence of acute renal failure and chronic kidney disease was found to be 2–3 times higher compared to the non-sickle group in a retrospective study (9). Renal impairment is a risk for drug toxicity as well as decreased drug efficacy.

The data about the safety and efficacy of voxelotor in chronic kidney disease is limited. Herein we report a case of a 49-year-old man, a known case of sickle cell disease and stage IV chronic kidney disease who was requiring frequent transfusion and suffering from severe body pain, managed successfully with voxelotor without affecting kidney function.

## Case report

A patient is a 49-year-old man with SCD (hemoglobin S/D double heterozygous), diabetes mellitus, and hypertension. His clinical course was complicated by chronic kidney stage IV, due to sickle glomerulopathy (baseline creatinine 250–300 umol/L and estimated GFR around 25 ml/min/1.73 m^2^), splenectomy at age 20, osteonecrosis of the left femoral head status post left hip replacement at age of 25. The patient required recurrent hospital visits for vaso-occlusive pain crises and blood transfusions despite being compliant with hydroxyurea 1000 mg twice daily.

**FIGURE 1:**
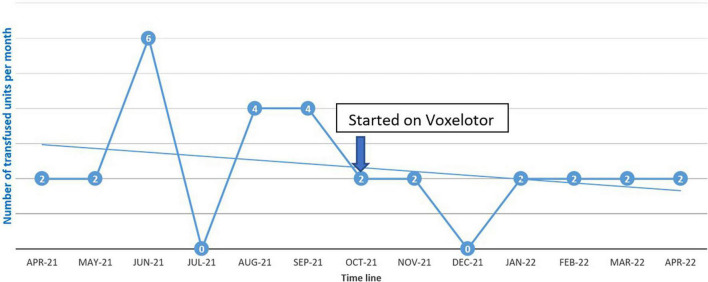
Transfusion requirement before and after starting voxelotor.

The patient was started on voxelotor 1,500 mg daily. After starting voxelotor, transfusion frequency decreased from every 2 weeks to every 4 weeks to maintain hemoglobin above 7 g/dL as can be seen in Figure 1. The patient reported that his body pain decreased by more than 70% and he did not report any of the known voxelotor side effects such as headache, nausea, diarrhea, or abdominal pain.

Close follow-up of kidney function over 6 months on voxelotor showed that creatinine and GFR remained stable, as seen in Table 1.

**TABLE 1:** Laboratory results 3 months before, at starting voxelotor, 3 months, and 6 months after.

Time	3 months	0 months	3 months	6 months
	before		after	after
Creatinine (umol/L)	262	255	264	251
eGFR (ml/min/1.73 m^2^)	25	26	25	26
Hemoglobin (gm/dl)	6.2	6.8	7.6	7.8
Red blood cells (×10^12^/L)	2.2	2.4	2.6	2.9
Hematocrit (%)	19.2	19.8	22.5	23.3
White blood cells (×10^9^/L)	8.2	8.8	9.7	7.9
Platelets (×10^9^/L)	151	166	157	170
LDH (U/L)	527	440	516	365
T. bilirubin (μmol/L)	34	39.5	24	22
D. bilirubin (μmol/L)	19	9		

eGFR, estimated glomerular filtration rate; LDH, lactate dehydrogenase; T. bilirubin, total bilirubin; D. bilirubin, direct bilirubin.

## Discussion

Polymerization of the deoxygenated sickle hemoglobin is the main driver of the pathophysiology of sickle cell disease. Voxelotor acts as a hemoglobin modulator by increasing its affinity to oxygen which prevents red blood cells from sickling. Voxelotor received FDA approval in late 2019 for sickle cell disease treatment following a multicenter, phase 3, double-blind, randomized, placebo-controlled trial. This trial showed that voxelotor provided a sustained increase in hemoglobin and a decrease in hemolytic markers (10, 11).

Pharmacokinetics and pharmacodynamics studies of voxelotor in healthy people have shown it is a rapidly absorbed drug, has an oral bioavailability of 35%, binds to protein at 99.8%, and its post-peak primarily metabolism in the liver. Hepatic metabolism is *via* phase I (oxidation and reduction) and phase II (glucuronidation). Around two-thirds of voxelotor and its metabolites are excreted in feces (62.6%) while around one-third are excreted in the urine (35.4%) after oral administration (12).

The data about the safety and efficacy of voxelotor in chronic kidney disease is limited. Preston et al. reported the only study in this regard (13). Its two open-label, parallel-group, phase I clinical trials, studied the safety and pharmacokinetics of voxelotor in patients with liver or kidney impairment. Considering that impaired kidney function may change voxelotor exposure, a total of eight patients with SCD and chronic kidney disease (estimated GFR < 30 mL/min/1.73 m2) and eight healthy, matched controls were given voxelotor 900 mg daily following an overnight fast. Subjects with normal kidney function were matched to patients with SCD having severe kidney impairment based on age, sex, and body mass index. Mean creatinine was 394 μmol/L and creatinine clearance was 12.5 ml/min in the renal impairment group. End-stage renal disease patients on dialysis were excluded. The result showed no apparent effect of renal function on voxelotor excretion on the basis of similar post-peak mean plasma voxelotor concentration decline between the two groups. There were no serious adverse events (AE), mortality, or medication discontinuation due to AE. Based on these results, Preston et al. (13) concluded that; voxelotor is safe and tolerable in patients with SCD along with severe chronic kidney disease and no dose adjustment is needed.

In our case, the patient had a poor quality of life due to recurrent hospital visits for blood transfusion and a vaso-occlusive painful crisis. After starting a 1500 mg daily dose of voxelotor, his transfusion requirement decreased, and he had a subjective decrease in body pain. In the Preston study, they used the dose of 900 mg daily as it was expected to be within the upper range of the therapeutic dose and was well tolerated in healthy subjects, however, we used the dose of 1,500 mg daily based on the HOPE trial which showed a higher hemoglobin response compared to the dose of 900 mg and pl acebo (7). Indices of kidney function remained stable 6 months after starting treatment which supports the previously reported study. Up to our knowledge, this is the first real-world reported case of voxelotor use in sickle cell disease patients with stage IV chronic kidney disease with the approved dose of voxelotor 1500 mg daily.

## Conclusion

In conclusion, considering the limited data, this case report indicates that voxelotor is safe and tolerable, and dose adjustment is not required in patients with SCD along with severe renal impairment; however, further studies are required to confirm this finding.

## Data availability statement

The original contributions presented in this study are included in the article/supplementary material, further inquiries can be directed to the corresponding author.

## Ethics statement

The studies involving human participants were reviewed and approved by the Medical Research Council, Hamad Medical Corporation. The patients/participants provided their written informed consent to participate in this case study. Written informed consent was obtained from the individual(s) for the publication of any potentially identifiable images or data included in this article.

## Author contributions

AA contributed to acquisition of data and drafted the manuscript. Both authors equally contributed to writing and editing.
